# Primary squamous cell carcinomas in the thyroid gland: an individual participant data meta-analysis

**DOI:** 10.1002/cam4.287

**Published:** 2014-07-04

**Authors:** Jae Keun Cho, Seung-Hoon Woo, Junoh Park, Min-Ji Kim, Han-Sin Jeong

**Affiliations:** 1 Department of Otorhinolaryngology-Head and Neck Surgery, Samsung Medical Center, Sungkyunkwan University School of MedicineSeoul, Korea; 2 Department of Otolaryngology, Gyeongsang National UniversityJinju, Korea; 3 Biostatistics and Clinical Epidemiology Center, Research Institute for Future Medicine, Samsung Medical CenterSeoul, Korea

**Keywords:** Diagnosis, outcomes, squamous cell carcinomas, thyroid gland, treatment

## Abstract

Primary squamous cell carcinomas arising from the thyroid gland (SCCTh) is extremely rare diseases, which have never been fully studied. Thus, we performed a systematic review and individual participant data meta-analysis of published SCCTh cases, to understand the clinical characteristics and to identify the prognostic factors of primary SCCTh. A literature search was conducted within Medline, EMBASE, Cochrane library databases and KoreaMed using the following Medical Subject Headings (MeSH) keywords: “primary,” “squamous,” “carcinoma,” “cancer,” and “thyroid.” Eighty-four patients' individual data from 39 articles and five patients' data in our institute were selected for analysis (*N* = 89). The mean age at diagnosis was 63.0 years (range, 24–90) and female preponderance (M:F = 1:2) was noted. The commonest complaint was the anterior neck mass, followed by dyspnea or dysphagia, and extension to the adjacent structure was found in 72%. The median survival was 9.0 months (95% CI, 6.0–23.0) and 3-year survival rate (3YSR) was 37.6% by Kaplan–Meier method, but only 20.1% by a shared frailty model for adjusting heterogeneity. Complete resection (R0) of tumors was the only significant prognostic factor in multivariable analysis, and the benefit of adjuvant treatment was not proved. The prognosis of patients with SCCTh is very poor (20% in 3YSR), but complete resection of disease is correlated with improved survival. To achieve complete surgical eradication of tumors, early detection and accurate diagnosis should be emphasized.

## Introduction

Primary squamous cell carcinoma in the thyroid gland (SCCTh) is a very rare malignant disease with an incidence of less than 1% of thyroid malignancies 1. Clinical characteristics and optimal treatment strategy of SCCTh are poorly defined because of their rarity 2. Most of the published articles reported experiences in a few cases, and there have been no comprehensive and systematic review except one literature review 3. Thus, we conducted a systematic review and individual participant data (IPD) meta-analysis focusing on the diagnosis, clinical courses and treatment outcomes of primary SCCTh. IPD meta-analysis reanalyzes the original research data, rather than extracting summary data from publications or investigators, which can improve the quality of data and produce more reliable results 4,5.

In a meta-analysis combining survival data from different clinical studies, an important issue is the possibility of heterogeneity between studies 6. In this situation, the shared frailty model, a method designed to account for variability due to unobserved individual-level factors, can combine study results by reducing the bias from the between-study variability 7. Thus, we applied two different methods; the Kaplan–Meier method and the shared frailty model to estimate overall survival rate and to identify risk factors from compiled data.

The aims of this study were to review the clinical characteristics of primary SCCTh, to identify the prognostic factors, and finally to suggest the proper treatment strategy for better clinical outcome. Especially we focused on resection status through surgical treatment with/without other adjuvant options. The results will help physicians predict prognosis and improve care for patients having SCCTh.

## Materials and Methods

### Search strategy

We conducted an electronic search of the paper in Medline database, EMBASE, and Cochrane library by recently updated articles (from 1981 to 2012). The search was restricted to English language in the abstract. Search term combinations were “primary,” “squamous,” “carcinoma,” “cancer,” and “thyroid.” In addition, we searched relevant studies in KoreaMed and hand-searched citations from the retrieved literature. With approval from our institutional review board we also conducted a retrospective review of medical records in our institute.

### Inclusion and exclusion of literatures

**Figure 1:**
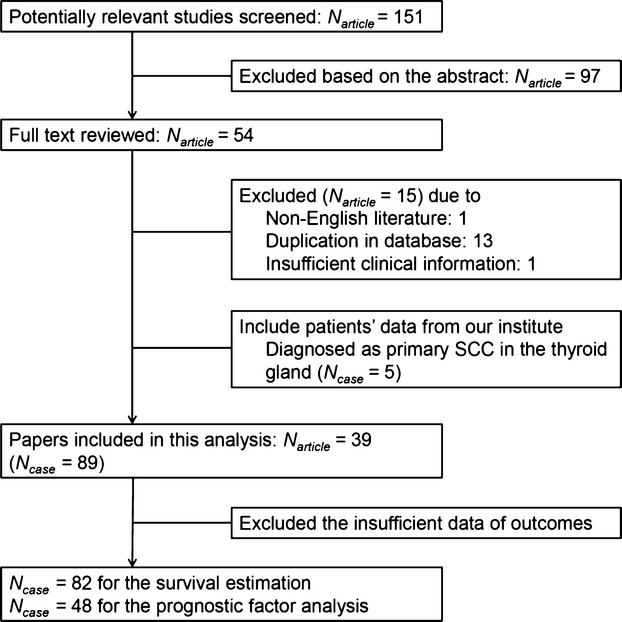
Literature search and study diagram.

We excluded nonenglish articles and studies that did not report sufficient clinical information. Duplication of data was ruled out by examining the names of all authors and their affiliations in each publication. We included published literatures in KoreaMed and patients' data from our institute who were diagnosed as primary SCCTh (Fig.1).

In all cases, physical examination of the aero-digestive tract was performed to exclude squamous cell carcinoma (SCC) from other origin sites in the head and neck area or metastasis to the thyroid gland. Radiological evaluations including chest CT, abdominal CT, or positron emission tomography (PET) or combined PET with CT (PET/CT) was also performed for the same purpose. All patients were finally diagnosed as primary SCCTh by surgically obtained specimens or autopsies, and in most of cases, final pathological diagnosis was confirmed with comprehensive pathologic review and immunohistochemical staining for differential diagnosis (Table S1). Variable markers were used including antibodies to low-molecular weight cytokeratin, thyroid transcription factor (TTF)-1, thyroglobulin, and calcitonin.

### Data extraction

Two investigators (J. K. C. and S. H. W.) reviewed each report for eligibility and extracted data from included studies. If the obtained data from literatures was insufficient or unclear for analysis, we sent an e-mail to each author appending with case report form in order to request complete data of mentioned patients. Patient and disease characteristics, including demographic data, age of diagnosis, gender, country (region), duration of symptoms, results of fine needle aspiration cytology (FNAC), disease extent, and treatment modalities were extracted from each literature and medical records. Among them, age at diagnosis, gender, disease extent, lymph node status, and treatment were evaluated for prognostic significance. In addition, the status of the resection margin was included in cases where pathological reports were available.

The main treatment modality was the surgical removal of tumors and the thyroid gland with or without neck dissection. External beam radiotherapy was targeted to the thyroid gland lesions and lower neck lymph nodes around the thyroid gland if needed clinically. Chemotherapy as a palliative treatment to the inoperable cases and immunotherapy were included as other treatment options.

Disease extent was divided into three categories: (1) the lesions limited to the thyroid or any tumor with minimal thyroidal extension, (2) tumor of any size extending beyond the thyroid capsule to invade subcutaneous soft tissues, larynx, trachea, esophagus, or recurrent laryngeal nerve, and (3) the disease with distant metastasis at diagnosis 8. The status of regional lymph nodes was recorded as no lymph node metastasis (N0) or the presence of lymph node metastasis (N1).

### Statistical analyses

The survival rate was estimated by Kaplan–Meier method, and the prognostic significance of clinical variables was evaluated by univariate and multivariable analysis. Age, gender, T stage (T1-3, T4), N stage (negative, positive), treatment (surgery only, surgery + adjuvant treatment, radiation or chemo-radiation), and resection status (complete R0 or incomplete R1) were considered as the variables. Second, we adjusted for the heterogeneity across the literature by using a shared frailty model with random trial effect 6,9. For this purpose, we chose studies including two or more patients' data and analyzed only in the cases where results of clinical variables were available.

In addition, the variables were compared between R0 and R1 using t-test for continuous variable (age) and chi-square test or Fisher's exact test for categorical variables. Findings were considered statistically significant with *P* < 0.05. R package, frailty pack (R 2.13.2, Vienna, Austria; http://www.R-project.org/) was used for fitting a shared frailty model and SAS version 9.3 (SAS Institute, Cary, NC) was used for the other statistical analysis.

## Results

### Subject enrollment

A total of 151 titles and abstracts were obtained through database searches. In these titles and abstracts, 54 full-text papers were deemed to be relevant and were examined in detail. After review, 39 papers were selected by the inclusion/exclusion criteria and these papers contributed information for 84 patients with primary SCCTh (Table S1). We also included five patients of primary SCCTh from our database in the IPD meta-analysis. Among 89 patients' data that were conducted comprehensive review, we performed survival analysis in 82 patients who had clinical information of survival outcome including our cases.

### Characteristics of patients and diseases (*N* = 89)

The mean age at diagnosis for 89 eligible patients was 63.0 years (range from 24 to 90) and there were 30 males and 59 females. The mean follow-up period was 16.9 months (range from 1 to 96). According to selected papers, the majority of patients' residential area was East Asia (52.8%). Only 20 patients (22.5%) had squamous cell carcinoma confined to thyroid gland, whereas 63 patients (70.8%) had extra-thyroidal extension to the adjacent organs, and six patients (6.7%) had distant metastasis at diagnosis. A total of 46 patients (51.7%) had no regional lymph node metastasis and 43 patients (48.3%) had regional lymph node metastasis. Subject and disease characteristics are presented in Table 1.

**Table 1:** Subject characteristics

*N* = 89	No. (%)
Age at diagnosis, (years) mean (range)	63.0 (24–90)
Gender	
Male: female	30:59 (33.7:66.3)
Region of reports	
North America	20 (22.5)
Europe	9 (10.1)
East Asia	47 (52.8)
Others	13 (14.6)
Disease extent	
Confined to thyroid gland	20 (22.5)
Extension to the adjacent organs	63 (70.8)
Distant metastasis	6 (6.7)
Lymph node involvement	
N0	46 (51.7)
N1	43 (48.3)
TNM stage	
T1-3N0M0	14 (15.7)
T4N0M0	32 (36.0)
T1-3N1M0	7 (7.9)
T4N1M0	30 (33.7)
M1	6 (6.7)
Treatment (*N* = 79)^1^	
Surgery alone	24 (30.4)
Surgery + Radiation	34 (43.0)
Surgery + chemo-radiation	8 (10.1)
Others (chemo or immunotherapy)	9 (11.4)
Palliative treatment	4 (5.1)
Final status (*N* = 82)^1^	
NED	26 (31.7)
AWD§	7 (8.5)
DOD	49 (59.8)
Recurrence sites reported (*N* = 16)^1^	
Loco-regional recur	7 (43.8)
Lung	3 (18.8)
Bone	4 (25.0)
Liver	1 (6.2)
Heart	1 (6.2)
Follow-ups (*N* = 82)^1^ (mean, range, months)	16.98 (1–96)

AWD, alive with disease; DOD, death of disease; NED, no evidence of disease.

1 Number of patients that was used in the analysis through gathering proper information from literatures or authors' reply.

§ Three patients had distant metastases (bone, *N* = 1 and lung, *N* = 2) at the last follow-ups (33–48 months).

### Initial symptoms (*N* = 60) and FNAC results (*N* = 59)

Most patients presented with enlarging neck mass or swelling (60.0%). Other patients complained of dyspnea/dysphagia (20.0%) and voice change (15.0%). Two patients had no initial symptoms and were diagnosed as SCCTh with screening ultrasonography with FNAC. FNAC was performed initially to evaluate mass lesion. In 41% of patients, FNAC results showed papillary thyroid carcinoma. High-grade malignancy such as poorly differentiated, undifferentiated or anaplastic thyroid carcinoma was suspected in 17%, and 27% of FNAC results predicted SCC or suspicious SCC. Meanwhile, 15% of FNAC results were nondiagnostic.

### Treatment and outcomes (*N* = 82)

Twenty-four patients (30.4%) with primary SCCTh underwent surgery alone, and 34 patients (43.0%) underwent surgical resection with adjuvant radiation therapy. Chemo-radiation after surgery was employed in eight patients (10.1%) and nine patients (11.4%) received other treatment such as upfront chemotherapy or immunotherapy. In four patients (5.1%), only palliative treatments including debulking surgery were used in order to secure airway.

**Figure 2:**
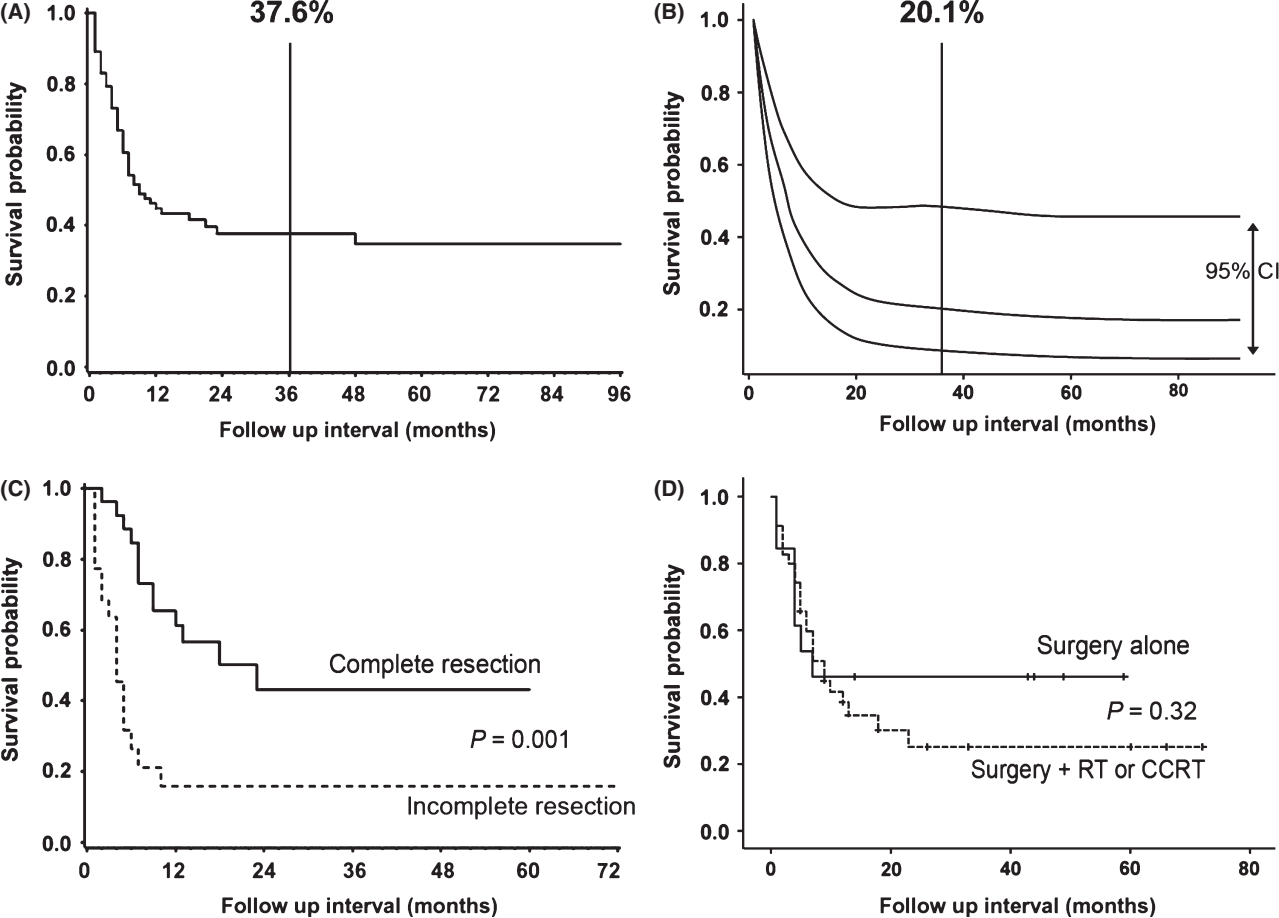
Survival plots. (A) Estimated survival rate of total subjects with primary squamous cell carcinomas in the thyroid gland by Kaplan–Meier method (*N* = 82). (B) Estimated survival rate of total subjects with primary SCC in the thyroid gland by shared frailty model for considering heterogeneity (*N* = 82). Upper and lower lines indicate 95% confidence interval. (C) Estimated survival rate of two groups: complete resection (R0) versus incomplete resection (R1) groups by Kaplan–Meier method (*N* = 48). (D) Survival curves of two groups: surgery alone versus surgery + postoperative adjuvant treatment (*N* = 48). RT, radiation therapy; CCRT, concurrent chemo-radiation; SCC, squamous cell carcinoma.

In their clinical courses, we could verify 49 patients who had died with disease. Among them, 36 patients (73.4%) were reported as having died with disease-specific causes. In the remaining 13 subjects, the cause of death was not clearly presented, however, we also considered them as disease-specific deaths, because the rapid progression of disease and deaths were stated in the articles. Loco-regional recurrences occurred in seven and distant metastases were reported in nine. Most common site of distant metastasis was bone (four patients, 25.0%) (Table 1). When the primary event of interest was overall survival, the median survival time was 9.0 months (95% CI, 6.0–23.0). Estimated survival rate at 3 years by Kaplan–Meier method was 37.6% and by a shared frailty model for considering heterogeneity was 20.1% (Fig.2A–B).

### Prognostic indicators from multivariable analysis (*N* = 48)

On univariate analysis, age at diagnosis, the extent of disease (T1-3 vs. T4), and resection margin status were prognostic for overall survival (Table 2). On multivariable analysis, resection margin status via surgical treatment and patient age were prognostic for survival. Particularly, securing adequate resection margin during operation was significantly effective for better survival outcome.

**Table 2:** The results of univariate and multivariable analysis for prognostic factors among clinical variables (*N* = 48)

	Univariate analysis		Multivariable analysis	
		
	Hazard ratio (95% CI)	*P*-value^1^	Hazard ratio (95% CI)	*P*-value^1^
Age	1.07 (1.03–1.11)	<0.001	1.06 (1.02–1.10)	0.006
M:F	0.55 (0.22–1.39)	0.209	0.38 (0.12–1.26)	0.113
T (T4/T1-3)	4.43 (1.28–15.33)	0.018	2.10 (0.37–11.83)	0.400
N (N1/N0)	2.59 (0.85–7.88)	0.092	1.18 (0.31–4.50)	0.805
Surgery + adjuvant Tx. versus surgery	1.56 (0.65–3.77)	0.320	0.80 (0.22–2.93)	0.734
R1/R0 resection	4.03 (1.71–9.48)	0.001	3.35 (1.09–10.35)	0.035

1 Analysis using a shared frailty model.

Comparing the two groups; R0 (complete resection of tumor) versus R1 (fail to remove completely tumors), R0 status had an advantage for survival (a shared frailty model, *P* = 0.035) and there was an absolute between-group difference with respect to rate of death from disease-specific cause, corresponding to a relative risk of incomplete/complete resection of 3.35 (95% CI: 1.09–10.35). Age was also a significant variable for survival outcome (a relative risk by increasing every single age of 1.06, 95% CI: 1.02–1.10, *P* = 0.006).

### Complete resection versus incomplete resection

Next, we checked the correlation between resection status and other variables (Table 3). Interestingly, the area where the patient lived was the only significant factor (*P* = 0.001). The extent of disease seemed to have some advantages to secure complete resection margin via surgical treatment (*P* = 0.066). Depending on the extent of diseases, patients had received thyroidectomy or wide surgical resection of tumors with/without neck dissection. Among these patients, 26 had negative resection margin and 22 patients had no adequate resection margin through surgical treatment. Patients with R0 resection had a 3-year survival rate (3YSR) of 43.1% and median survival time of 23 months. However, R1 resection was associated with a 3YSR of 15.9% and median survival time of 4 months (Fig.2C).

**Table 3:** Association with complete resection and the confounders (*N* = 48)

	R0 resection (*N* = 26)	R1 resection (*N* = 22)	
	No. (%)	No. (%)	*P*-value
Mean follow-up time (range, months)	18.42 (2–60)	11.73 (1–72)	0.875^1^
Mean age (±SD)	61.9 (±13.5)	62.7 (±13.4)	
Gender			
Male	11 (42.3)	8 (36.4)	0.674^2^
Female	15 (57.7)	14 (63.6)	
Area			
Asian	25 (96.1)	12 (54.6)	0.001^2^
Western	1 (3.9)	10 (45.4)	
Disease extent			
Confined to thyroid	8 (30.8)	2 (9.1)	0.066^3^
Extension to adjacent structure or distant metastasis	18 (69.2)	20 (90.9)	
Lymph node involvement			
Yes	15 (57.7)	12 (54.6)	0.724^3^
No	11 (42.3)	10 (45.4)	
Treatment			
Surgery	7 (26.9)	6 (27.3)	0.978^3^
Surgery + adjuvant Tx.	19 (73.1)	16 (72.7)	

1 Independent-samples *T*-test.

2 Chi-square test.

3 Fisher's exact test.

## Discussion

Primary SCCTh is extremely rare, and disease characteristics and treatment outcomes of SCCTh are not well established, although SCCTh usually has an aggressive clinical course 1. Due to this aggressiveness, treatment failure frequently occurs within 6 months after an initial treatment 10–12. In several studies based on small numbers of patients with primary SCC in the thyroid gland, early diagnosis and extensive surgical treatment with/without adjuvant multi-treatment modalities were recommended 3,13,14, which are confirmed by our analyses.

Most important differential diagnosis of primary SCCTh are carcinoma showing thymus-like elements (CASTLE) disease of the thyroid gland, anaplastic thyroid carcinoma, and metastasis from adjacent upper aero-digestive organs 15. In addition, the association between SCCTh and tall-cell variant of papillary carcinoma has been reported 16,17. The histopathologic diagnosis of primary SCCTh requires microscopic identification of keratin or intercellular bridge structures 11,16,18. Also, squamoid differentiation in an anaplastic thyroid carcinoma are similar to nonkeratinizing SCC 19, but in cases of primary SCCTh, there were no giant cells, syncythial elements, or atypical mitoses features 20,21. In most of cases, final pathological diagnosis was confirmed with comprehensive pathologic review and immunohistochemical staining for differential diagnosis, such as cytokeratin, TTF-1, thyroglobulin, and calcitonin 22,23.

In addition, one should keep it mind the possibility from metastasis or secondary involvement of adjacent organ in the head and neck area. When the thyroid mass lesion reveals as SCC pathology, it is known that cases of metastasis or direct invasion of SCC into the thyroid gland are more common than primary SCCTh 24. Definitive diagnosis of primary SCC in the thyroid gland have to be made only after comprehensive clinical investigation for excluding direct invasion from adjacent organs or metastasis from SCC in other distant organs 13,25. We reviewed all selected papers and found that every investigator had performed physical examination with full endoscope and proper radiologic evaluations.

Cytological evidence of primary SCCTh cannot be easily detected by FNAC. In our study, the predictability of diagnosis in FNAC is inferior to one-third of patients. More than half of cases had been diagnosed as papillary thyroid carcinoma or were nondiagnostic. Conversely, in 40% of diseases FNAC suggested high-grade features on smear. Thus, FNAC suspicious of high-grade malignancy combined with clinical/radiological findings can be the most important diagnostic clue for primary SCCTh. Even though, there were no clear data about core needle biopsy in our literature review, core needle biopsy should be considered for proper diagnosis, in highly suspicious cases.

Because of disease rarity, there was no consensus of treatment modality or the extent of surgery. We performed the subgroup analysis to evaluate proper surgical extent (hemi-thyroidectomy vs. total thyroidectomy) in R0 resection cases. There were no clinical significances between two types of thyroidectomy groups (HR = 0.36 [95% CI, 0.09–1.34], *P* = 0.126). After surgery, the additional adjuvant treatments were also fitted in each case. However, in advanced stage disease, the success rate of radical surgical resection was compromised by the invasive and infiltrative nature of SCC. Therefore, it is very challenging to determine the extent of surgical resection, which needs further study.

Primary SCCTh is relatively resistant to radiotherapy and poorly responsive to chemotherapy 14,26, thus complete resection through surgical treatment may improve survival 13. In this study, we could ascertain the only benefit for survival in case of complete resection. However, complete resection is a surrogate for tumor extent and local invasiveness. These factors likely account for a large part of the improved prognosis of completely resected tumors. In addition, the benefits from addition of radiation therapy or chemo-radiation therapy following surgical treatment were not evaluated precisely in our analysis, since reviewed papers did not provide information about radiation dose, technique, fractionation or chemotherapy (Fig.2D). The majority of published studies carefully concluded that the effect of postoperative adjuvant treatment may help to achieve better outcome in certain cases and anaplastic thyroid carcinomas 27. In our analysis, there was lack of additional information about loco-regional or distant metastasis after treatment that was mainly affected disease prognosis. This is a limitation of our study, and further evaluation should be performed.

In this study, we used the Kaplan–Meier method, and the shared frailty model to estimate overall survival rate and to identify risk factors. The estimated survival by Kaplan–Meier method tended to be overestimated compared to the estimated baseline survival by the shared frailty model considering heterogeneity between literatures. Despite of our efforts to reduce the bias from heterogeneity, the lack of enough cases was another limitation with this study. However, to the best of our knowledge, this study is the first integrated investigation of treatment outcome and prognostic factors in primary SCCTh.

## Conclusion

The prognosis of patients with SCCTh is very poor (20% in 3YSR), but complete resection of disease was correlated with improved survival. To achieve safe eradication of tumors, early detection and accurate diagnosis should be emphasized for better prognosis.

## Supporting Information

**Table S1.** Individual participant data from enrolled cases reported previously as primary squamous cell carcinoma in the thyroid gland and our series.
