# Supplementary material for: Primary squamous cell carcinomas in the thyroid gland: an individual participant data meta-analysis
Source: Cancer Med. 2014 Jul 4;3(5):1396–403. doi: 10.1002/cam4.287 (PMC4302690; doi:10.1002/cam4.287)
Supplement: Supplementary file 1 — Table S1. Individual participant data from enrolled cases reported previously as primary squamous cell carcinoma in the thyroid gland and our series. [file cam40003-1396-SD1.docx]

**SUPPLEMENTARY TABLE 1.** Individual Participant Data from Enrolled Cases Reported Previously As Primary Squamous Cell Carcinoma in the Thyroid Gland and Our Series

| No. | Authors | Age/Sex | Area | TNM stage | Treatment | Operation | Resection Status | IHC positive | IHC negative | Survival/FU(mo) |
| --- | --- | --- | --- | --- | --- | --- | --- | --- | --- | --- |
| 1 | Tunio, M.A. et al.[^1^](#_ENREF_1) | 54/F | West South Asia | T4bN0M0 | RT | - | R1 | CK5/6, DK903, ki67(30%), p53(30%) | CK20, Tg, calcitonin, synaptophysin, mucidarmin | DOD/3 |
| 2 | Shrestha M. et al.[^2^](#_ENREF_2) | 75/F | North America | T4aN0M0 | OP+RT | TT+ND+TL | R0 | TTF-1, p53 |  | DOD/21 |
| 3 | Mercante G. et al.[^3^](#_ENREF_3) | 67/M | East Asia | T4aN0M0 | OP+CCRT, RAI | Debulking | R1 | p63 | TTF-1, CD5 | AWD/48 |
| 4 | Chen KH. et al.[^4^](#_ENREF_4) | 67/F | East Asia | T3aN1bM0 | OP+RT | HT+CND | R0 | CK5/6, CK19, p63, EMA, p53 | TTF-1, Tg, galectin3, CD5 | DOD/7 |
| 5 | Ko YS. et al.[^5^](#_ENREF_5) | 87/M | East Asia | T4aN1bM0 | OP+RT | TT+ND | R0 |  |  | DOD/11 |
| 6 | Ito Y. et al.[^6^](#_ENREF_6) | 71/F | East Asia | T4aN1bM0 | IC, OP | TT+ND | R0 |  |  | DOD/18 |
| 7 | Ito Y. et al.[^6^](#_ENREF_6) | 71/F | East Asia | T1N1bM0 | OP | TT+ND | R0 |  |  | NED/43 |
| 8 | Ito Y. et al.[^6^](#_ENREF_6) | 69/F | East Asia | T1N1bM1 | OP+CT | TT+CND | R0 |  |  | AWD/33 |
| 9 | Ito Y. et al.[^6^](#_ENREF_6) | 67/F | East Asia | T4aN0M0 | IC, OP | TT+ND | R0 |  |  | DOD/23 |
| 10 | Ito Y. et al.[^6^](#_ENREF_6) | 83/F | East Asia | T4aN1aM0 | OP+CCRT | TT+ND | R0 |  |  | NED/18 |
| 11 | Ito Y. et al.[^6^](#_ENREF_6) | 76/M | East Asia | T4aN1aM0 | OP | TT+ND | R0 |  |  | NED/14 |
| 12 | Ito Y. et al.[^6^](#_ENREF_6) | 71/M | East Asia | T4aN1aM0 | OP+RT | TT+ND | R0 |  |  | NED/13 |
| 13 | Ito Y. et al.[^6^](#_ENREF_6) | 68/F | East Asia | T4aN1aM0 | IC, OP+RT | HT+ND | R0 |  |  | NED/9 |
| 14 | Ito Y. et al.[^6^](#_ENREF_6) | 70/M | East Asia | T4aN1aM0 | OP, IT | TT+ND | R0 |  |  | DOD/9 |
| 15 | Ito Y. et al.[^6^](#_ENREF_6) | 71/F | East Asia | T4aN1aM0 | OP+CT | TT+ND | R1 |  |  | NED/5 |
| 16 | Chen CY. et al. ^§^[^7^](#_ENREF_7) | 60/F | East Asia | T3N0M0 | OP+RT | TT | R0 | CK5/6 | TTF-1, CD5 | -/- |
| 17 | De Vos FY. et al.[^8^](#_ENREF_8) | 51/F | Europe | T4aN0M0 | IC, OP+RT. | HT+ND | R0 |  |  | NED/20 |
| 18 | Yucel H. et al.[^9^](#_ENREF_9) | 88/M | Europe | T4aN0M0 | OP+RT. | TT | R0 |  |  | NED/6 |
| 19 | Rausch T. et al.[^10^](#_ENREF_10) | 82/M | Europe | T4aN0M0 | OP | HT+ND | R1 | CK5/6, p63, p53, TTF-1, Tg, MIB-1(5%) |  | DOD/2 |
| 20 | Long JL. et al.[^11^](#_ENREF_11) | 57/M | North America | T3N1bM0 | OP+CCRT | TT+ND | R0 | EGFR |  | NED/12 |
| 21 | Eom TI. et al.[^12^](#_ENREF_12) | 43/F | East Asia | T3N0M0 | OP+RT, RAI | TT | R0 | p63, galectin-3 | Tg, calcitonin | NED/8 |
| 22 | Makay O. et al.[^13^](#_ENREF_13) | 53/M | Europe | T3N0M0 | OP+RT | TT | R0 | CK19, MIB-1(high) | Tg, calcitonin, CD5 | DOC/2 |
| 23 | Makay O. et al.[^13^](#_ENREF_13) | 71/M | Europe | T4aN1bM0 | OP | Debulking | R1 | CK19, MIB-1(high) | Tg, calcitonin, CD5 | DOD/4 |
| 24 | Makay O. et al.[^13^](#_ENREF_13) | 66/M | Europe | T4aN0M0 | OP | TT | R1 | CK19, MIB-1(high) | Tg, calcitonin, CD5 | DOD/5 |
| 25 | Müssig K. et al. ^§^[^14^](#_ENREF_14) | 66/M | South Asia | T4aN1bM1 | OP | PR+ND | - | CK5/6, CK7, CK18, CK19, Ki67(20%) | TTF-1, Tg | -/- |
| 26 | Chintamani. et al.[^15^](#_ENREF_15) | 50/F | South Asia | T4aN1aM0 | OP+RT | HT+CND | R0 | pancytokeratin | Tg, calcitonin | DOD/7 |
| 27 | Chintamani. et al.[^15^](#_ENREF_15) | 60/M | South Asia | T4aN1bM0 | OP+RT | WR+ND | R0 |  |  | DOC/15 |
| 28 | Chintamani. et al.[^15^](#_ENREF_15) | 58/M | South Asia | T4aN1bM0 | OP+RT | WR+ND | R0 |  |  | NED/12 |
| 29 | Jung TS. et al.[^16^](#_ENREF_16) | 56/M | East Asia | T3N0M0 | OP+RT | TT | R0 | CK19 | TTF-1 | NED/96 |
| 30 | Kitahara S. et al.[^17^](#_ENREF_17) | 82/F | East Asia | T4aN1bM0 | OP | WR | - | MIB-1(43%), p53 |  | DOD/6 |
| 31 | Sutak J. et al. ^§^[^18^](#_ENREF_18) | 80/F | Europe | T4aN1bM0 | OP | TT+CND | R1 | pancytokeratin, CK7, CK19, p53 | TTF-1, Tg | -/- |
| 32 | Sahoo M. et al.[^19^](#_ENREF_19) | 42/F | South Asia | T3N1bM0 | OP | HT+ND | R1 | CK | Tg, calcitonin | DOD/6 |
| 33 | Sahoo M. et al.[^19^](#_ENREF_19) | 55/F | South Asia | T4aN1bM0 | OP | HT+ND | - | CK | calcitonin | DOD/3 |
| 34 | Zhou XH. et al.[^20^](#_ENREF_20) | 71/F | East Asia | T4aN0M0 | OP | TT | R0 | CK, EMA |  | DOD/4 |
| 35 | Zhou XH. et al.[^20^](#_ENREF_20) | 69/F | East Asia | T4aN0M0 | OP+RT | HT | R0 | CK, EMA |  | DOD/6 |
| 36 | Zhou XH. et al.[^20^](#_ENREF_20) | 63/F | East Asia | T4aN0M0 | OP+RT | TT | R0 | CK, EMA, Tg |  | DOD/13 |
| 37 | Zhou XH. et al.[^20^](#_ENREF_20) | 28/F | East Asia | T4aN0M0 | OP+CCRT | TT | R0 | CK, EMA |  | NED/26 |
| 38 | Lam KY. et al.[^21^](#_ENREF_21) | 82/F | East Asia | T4aN0M0 | OP+RT | Debulking | R1 | CK7, CK19, p53 |  | DOD/2 |
| 39 | Lam KY. et al.[^21^](#_ENREF_21) | 66/F | East Asia | T4aN1bM0 | OP | TT | R1 | CK19 |  | DOD/4 |
| 40 | Lam KY. et al.[^21^](#_ENREF_21) | 81/F | East Asia | T4aN1bM0 | - | - | - | CK7, CK19, p53 |  | DOD/0 |
| 41 | Lam KY. et al.[^21^](#_ENREF_21) | 56/F | East Asia | T4aNxMx | OP | Debulking | R1 | CK7, CK18, CK19 |  | DOD/0 |
| 42 | Jones JM. et al.[^22^](#_ENREF_22) | 48/M | North America | T4aN1bM0 | OP+RT | TT+ND | R0 |  | Tg, calcitonin | DOD/8 |
| 43 | Kleer CG. et al. ^§^[^23^](#_ENREF_23) | 64/M | North America | T4aN0M0 | - | TT+ND | - |  |  | -/- |
| 44 | Kleer CG. et al. ^§^[^23^](#_ENREF_23) | 66/F | North America | T4aN0M0 | - | TT+ND | - |  |  | -/- |
| 45 | Kleer CG. et al.[^23^](#_ENREF_23) | 38/M | North America | T4aN1bM0 | - | TT+ND | - |  |  | AWD/61 |
| 46 | Kleer CG. et al.[^23^](#_ENREF_23) | 90/F | North America | T4aN1bM0 | - | TT+ND | - |  |  | DOD/6 |
| 47 | Kleer CG. et al.[^23^](#_ENREF_23) | 74/F | North America | T4aN1bM0 | - | TT+ND | - |  |  | AWD/55 |
| 48 | Kleer CG. et al.[^23^](#_ENREF_23) | 78/F | North America | T4aN1M0 | - | TT+ND | - | p53 |  | AWD/54 |
| 49 | Kleer CG. et al.[^23^](#_ENREF_23) | 74/F | North America | T4aN1M0 | - | TT+ND | - | p53 |  | AWD/54 |
| 50 | Kleer CG. et al.[^23^](#_ENREF_23) | 50/F | North America | T4aN1bM1 | - | - | - |  |  | AWD/58 |
| 51 | Wan Muhaizan WM. et al.[^24^](#_ENREF_24) | 64/F | East South Asia | T3N0M0 | OP+RT | TT | R0 | CK | Tg | NED/24 |
| 52 | Batchelor N.K. et al.[^25^](#_ENREF_25) | 75/F | North America | T4aN0M0 | OP+RT | TT+ND+TL | R0 | CK5/6, CK903, Ki67(30%), p53(30%), TTF-1 | Tg, calcitonin, chromagranin | NED/15 |
| 53 | Ab Hadi I.S. et al.[^26^](#_ENREF_26) | 60/F | Europe | T3N0M0 | OP+RT | TT | R0 | CK |  | NED/- |
| 54 | Misonou J. et al. ^§^[^27^](#_ENREF_27) | 61/F | East-Asia | T4aN1bM0 | OP+RT | HT+ND | R1 |  |  | DOD/6 |
| 55 | Simpson W.J. et al.[^28^](#_ENREF_28) | 58/M | North America | T4bN0M0 | OP+RT | WR | R1 |  |  | NED/72 |
| 56 | Simpson W.J. et al.[^28^](#_ENREF_28) | 43/M | North America | T4aN0M0 | OP+RT | WR | R1 |  |  | NED/66 |
| 57 | Simpson W.J. et al.[^28^](#_ENREF_28) | 72/M | North America | T3N1bM0 | OP+RT | PR | R1 |  |  | DOD/1 |
| 58 | Simpson W.J. et al.[^28^](#_ENREF_28) | 65/M | North America | T4aN0M0 | OP+RT | PR | R1 |  |  | DOD/6 |
| 59 | Simpson W.J. et al.[^28^](#_ENREF_28) | 52/F | North America | T4bN0M0 | OP+CCRT | PR | R1 |  |  | DOD/5 |
| 60 | Simpson W.J. et al.[^28^](#_ENREF_28) | 62/F | North America | T4bN0M0 | OP | PR | R1 |  |  | DOD/1 |
| 61 | Simpson W.J. et al.[^28^](#_ENREF_28) | 72/M | North America | T4aN1bM1 | CCRT | - | R1 |  |  | DOD/1 |
| 62 | Simpson W.J. et al.[^28^](#_ENREF_28) | 58/F | North America | T4aN1bM0 | CCRT | - | R1 |  |  | DOD/4 |
| 63 | Kapoor V.K. et al.[^29^](#_ENREF_29) | 45/F | South Asia | T4bN0M0 | OP | Debulking | R1 |  |  | DOD/2 |
| 64 | Segal K. et al.[^30^](#_ENREF_30) | 47/F | West South Asia | T3N0M0 | OP | TT | R0 |  |  | NED/14 |
| 65 | Segal K. et al.[^30^](#_ENREF_30) | 63/M | West South Asia | T4aN0M0 | OP+RT | TT | R0 |  |  | NED/12 |
| 66 | Segal K. et al.[^30^](#_ENREF_30) | 73/F | West South Asia | T3N0M0 | OP+RT | TT+TL | R1 |  |  | DOD/1 |
| 67 | Segal K. et al.[^30^](#_ENREF_30) | 60/M | West South Asia | T4aN0M0 | OP+CCRT | Debulking | R1 |  |  | DOD/3 |
| 68 | Segal K. et al.[^30^](#_ENREF_30) | 61/F | West South Asia | T4aN0M0 | OP+RT | HT | R0 |  |  | DOD/5 |
| 69 | Segal K. et al.[^30^](#_ENREF_30) | 65/F | West South Asia | T4aN0M0 | OP+RT | WR | R1 |  |  | DOD/7 |
| 70 | Saito K. et al.[^31^](#_ENREF_31) | 71/F | East Asia | T4aN1bM1 | IC | - | R1 |  |  | DOD/1 |
| 71 | Choi JS. et al.[^32^](#_ENREF_32) | 24/F | East Asia | T1bN0M0 | OP | TT+ND | R1 |  |  | NED/49 |
| 72 | Choi JS. et al.[^32^](#_ENREF_32) | 84/F | East Asia | T4aN1bM0 | OP+RT | TT+ND | R1 |  |  | DOD/5 |
| 73 | Choi JS. et al.[^32^](#_ENREF_32) | 41/M | East Asia | T2N0M0 | OP | TT+CND | R0 |  |  | NED/59 |
| 74 | Choi JS. et al.[^32^](#_ENREF_32) | 55/F | East Asia | T4aN1bM0 | OP+CCRT | TT+ND | R1 |  |  | DOD/10 |
| 75 | Choi JS. et al.[^32^](#_ENREF_32) | 76/F | East Asia | T4aN0M0 | OP+RT | TT+CND | R1 |  |  | DOD/4 |
| 76 | Choi JS. et al.[^32^](#_ENREF_32) | 33/F | East Asia | T1bN0M0 | OP | TT+CND | R0 |  |  | NED/49 |
| 77 | Kim HS. et al.[^33^](#_ENREF_33) | 30/F | East Asia | T1bN0M0 | OP | TT+CND | R0 |  |  | NED/20 |
| 78 | Kwak JH. et al.[^34^](#_ENREF_34) | 70/M | East Asia | T1bN0M0 | OP | TT | R0 |  | Tg | NED/24 |
| 79 | Joo YH. et al.[^35^](#_ENREF_35) | 69/M | East Asia | T4aN0M0 | OP+RT | TT+CND+ND | R0 |  | Tg | DOD/8 |
| 80 | Bae KI. et al.[^36^](#_ENREF_36) | 80/F | East Asia | T4aN0M0 | OP | Debulking | R1 |  |  | DOD/2 |
| 81 | Lee KW. et al.[^37^](#_ENREF_37) | 75/F | East Asia | T4aN1bM1 | OP+CCRT | PR | R1 |  |  | DOD/1 |
| 82 | Lee KW. et al.[^37^](#_ENREF_37) | 61/M | East Asia | T4aN1bM0 | - | PR | R1 |  |  | DOD/1 |
| 83 | Tae K. et al.[^38^](#_ENREF_38) | 58/F | East Asia | T4aN0M0 | OP+RT | TT+ND | R0 |  |  | DOD/5 |
| 84 | Yoon IJ. et al. ^§^[^39^](#_ENREF_39) | 43/F | East Asia | T3N0M0 | OP | TT | R0 | CK | Tg | -/- |
| 85 | Case 1^*^ | 55/M | East Asia | T2N0M0 | OP+RT | TT | R0 |  | Tg, calcitonin | NED/over 60 |
| 86 | Case 2^*^ | 72/F | East Asia | T2N0M0 | OP | HT | R0 |  |  | DOD/7 |
| 87 | Case 3^*^ | 50/F | East Asia | T4N0M0 | OP | PR | R1 |  |  | DOD/2 |
| 88 | Case 4^*^ | 79/M | East Asia | T4N0M0 | OP+RT | TT+TL | R0 |  | TTF-1, CD5 | DOD/9 |
| 89 | Case 5^*^ | 60/M | East Asia | T4N0M0 | IC, OP+RT | TT+ND | R0 | p63, TTF-1, Tg |  | DOD/7 |

^*^ Patients’ data extracted from retrospective medical records review in our institute

**^§^** Cases excluded survival analysis due to insufficient clinical outcome

OP, surgical treatment; OP+RT, surgical treatment and radiation therapy; OP+CCRT, surgical treatment and chemo-radiation therapy; IC, induction chemotherapy; CT, chemotherapy; IT, immunotherapy; RAI, radioactive iodine therapy; TT, total thyroidectomy; HT, hemi-thyroidectomy; TL, total laryngectomy; ND, neck dissection; CND, central neck dissection; WR, wide resection; PR, partial resection; R0, complete resection status; R1, incomplete resection status; DOD, death of disease; DOC, death of other cause; AWD, alive with disease; NED, no evidence of disease

**Supplementary References**

1. Tunio MA, Al Asiri M, Fagih M, Akasha R. Primary squamous cell carcinoma of thyroid: a case report and review of literature. *Head Neck Oncol.* 2012;4:8.

2. Shrestha M, Sridhara SK, Leo LJ, Coppit GL, 3rd, Ehrhardt NM. Primary squamous cell carcinoma of the thyroid gland: A case report and review. *Head Neck.* 2013;35:E299-303.

3. Mercante G, Marchesi A, Covello R, Dainese L, Spriano G. Mixed squamous cell carcinoma and follicular carcinoma of the thyroid gland. *Auris Nasus Larynx.* 2012;39:310-313.

4. Chen KH, Chou YH, Cheng AL. Primary squamous cell carcinoma of the thyroid with cardiac metastases and right ventricle outflow tract obstruction. *J Clin Oncol.* 2012;30:e260-263.

5. Ko YS, Hwang TS, Han HS, Lim SD, Kim WS, Oh SY. Primary pure squamous cell carcinoma of the thyroid: report and histogenic consideration of a case involving a BRAF mutation. *Pathol Int.* 2012;62:43-48.

6. Ito Y, Hirokawa M, Higashiyama T, et al. Biological behavior of papillary carcinoma of the thyroid including squamous cell carcinoma components and prognosis of patients who underwent locally curative surgery. *J Thyroid Res.* 2012;2012:230283.

7. Chen CY, Tseng HS, Lee CH, Chan WP. Primary squamous cell carcinoma of the thyroid gland with eggshell calcification: sonographic and computed tomographic findings. *J Ultrasound Med.* 2010;29:1667-1670.

8. De Vos FY, Sewnaik A, de Wilt JH, Smid EJ, den Bakker MA, van Meerten E. Combined therapy for thyroid squamous cell carcinoma. *Head Neck.* 2012;34:131-134.

9. Yucel H, Schaper NC, van Beek M, Bravenboer B. Primary squamous cell carcinoma of the thyroid years after radioactive iodine treatment. *Neth J Med.* 2010;68:224-226.

10. Rausch T, Benhattar J, Sutter M, Andrejevic-Blant S. Thyroid carcinoma with papillary and squamous features: report of a case with histogenetic considerations. *Pathol Res Pract.* 2010;206:263-269.

11. Long JL, Strocker AM, Wang MB, Blackwell KE. EGFR expression in primary squamous cell carcinoma of the thyroid. *Laryngoscope.* 2009;119:89-90.

12. Eom TI, Koo BY, Kim BS, et al. Coexistence of primary squamous cell carcinoma of thyroid with classic papillary thyroid carcinoma. *Pathol Int.* 2008;58:797-800.

13. Makay O, Kaya T, Ertan Y, et al. Primary squamous cell carcinoma of the thyroid: report of three cases. *Endocr J.* 2008;55:359-364.

14. Mussig K, Dahm J, Koitschev A, et al. Primary squamous cell carcinoma of the thyroid. *Intern Med J.* 2008;38:69-70.

15. Chintamani, Kulshreshtha P, Singh J, et al. Is an aggressive approach justified in the management of an aggressive cancer--the squamous cell carcinoma of thyroid? *Int Semin Surg Oncol.* 2007;4:8.

16. Jung TS, Oh YL, Min YK, et al. A patient with primary squamous cell carcinoma of the thyroid intermingled with follicular thyroid carcinoma that remains alive more than 8 years after diagnosis. *Korean J Intern Med.* 2006;21:73-78.

17. Kitahara S, Ito T, Hamatani S, Shibuya K, Shiba T. Thyroid papillary carcinoma recurring as squamous cell carcinoma: report of a case. *Surg Today.* 2006;36:171-174.

18. Sutak J, Armstrong JS, Rusby JE. Squamous cell carcinoma arising in a tall cell papillary carcinoma of the thyroid. *J Clin Pathol.* 2005;58:662-664.

19. Sahoo M, Bal CS, Bhatnagar D. Primary squamous-cell carcinoma of the thyroid gland: new evidence in support of follicular epithelial cell origin. *Diagn Cytopathol.* 2002;27:227-231.

20. Zhou XH. Primary squamous cell carcinoma of the thyroid. *Eur J Surg Oncol.* 2002;28:42-45.

21. Lam KY, Lo CY, Liu MC. Primary squamous cell carcinoma of the thyroid gland: an entity with aggressive clinical behaviour and distinctive cytokeratin expression profiles. *Histopathology.* 2001;39:279-286.

22. Jones JM, McCluggage WG, Russell CF. Primary squamous carcinoma of the thyroid. *Ulster Med J.* 2000;69:58-60.

23. Kleer CG, Giordano TJ, Merino MJ. Squamous cell carcinoma of the thyroid: an aggressive tumor associated with tall cell variant of papillary thyroid carcinoma. *Mod Pathol.* 2000;13:742-746.

24. Wan Muhaizan WM, Phang KS, Sharifah NA, al Amin D. Primary squamous cell carcinoma of the thyroid--a case report. *Malays J Pathol.* 1998;20:109-111.

25. Batchelor NK. Primary squamous cell carcinoma of the thyroid: an unusual presentation. *J Bronchology Interv Pulmonol.* 2011;18:168-170.

26. Ab Hadi I, Bliss RD, Lennard TW, Welch AR. Primary squamous cell carcinoma of the thyroid gland: a case report and role of radiotherapy. *Surgeon.* 2007;5:249-251.

27. Misonou J, Aizawa M, Kanda M, Uekita Y, Motohara T. Pure squamous cell carcinoma of the thyroid gland--report of an autopsy case and review of the literature. *Jpn J Surg.* 1988;18:469-474.

28. Simpson WJ, Carruthers J. Squamous cell carcinoma of the thyroid gland. *Am J Surg.* 1988;156:44-46.

29. Kapoor VK, Sharma D, Mukhopadhyay AK, Chattopadhyay TK. Primary squamous cell carcinoma of the thyroid gland--a case report. *Jpn J Surg.* 1985;15:60-62.

30. Segal K, Sidi J, Abraham A, Konichezky M, Ben-Bassat M. Pure squamous cell carcinoma and mixed adenosquamous cell carcinoma of the thyroid gland. *Head Neck Surg.* 1984;6:1035-1042.

31. Saito K, Kuratomi Y, Yamamoto K, et al. Primary squamous cell carcinoma of the thyroid associated with marked leukocytosis and hypercalcemia. *Cancer.* 1981;48:2080-2083.

32. Choi JS, Lim JY, Chu YC, Song SU, Kim YM. Analysis of primary squamous cell carcinoma of thyroid. *Korean J Otorhinolaryngol-Head Neck Surg.* 2012;55:26-29.

33. Kim HS, Hahm JR, Jung TS, et al. A case of mixed papillary thyroid tumor and squamous-cell carcinoma. *Yeungnam Univ J Med.* 2011;28:206-210.

34. Kwak JH, Jang HJ, Kim JH, Ahn JH, Kang KH, Han MS. A patient with mixed squamous cell and papillary thyroid carcinoma. *J Korean Surg Soc.* 2010;78:55-57.

35. Joo YH, Kim JH, Sun DI, Kim MS. A case of synchronous squamous cell and papillary carcinoma of the thyroid gland. *Korean J Otorhinolaryngol-Head Neck Surg.* 2008;51:1065-1067.

36. Bae KI, Kim KH, Yang SY, Lee SH, Kwon SK, Jung SJ. A case of primary squamous cell carcinoma of the thyroid gland. *J Korean Soc Endocrinol.* 2005;20:84-89.

37. Lee KW, Park SY, Lee SK, et al. Two cases with squamous cell carcinoma of the thyroid gland. *J Korean Soc Endocrinol.* 1998;13:446-452.

38. Tae K, Lee HS, Park JS, Jang SJ. A case of primary squamous cell carcinoma of the thyroid gland. *Korean J Otolaryngol-Head Neck Surg.* 1998;41:952-955.

39. Yoon IJ, Park ES, Yoo JH. Fine needle aspiration cytology of squamous cell carcinoma of the thyroid: report of a case. *Korean J Cytopathol.* 1997;8:57-61.
